# The Role of cGAS-STING-Driven PANoptosis in Neurodegenerative Diseases and Therapeutic Prospects

**DOI:** 10.3390/cells15181631

**Published:** 2026-09-09

**Authors:** Xinyi Hou, Wei Yu

**Affiliations:** 1 Institute of Biochemistry, College of Life Sciences and Medicine, Zhejiang Sci-Tech University, Hangzhou 310018, China; 2025210901030@mails.zstu.edu.cn; 2 Zhejiang Provincial Key Laboratory of Silkworm Bioreactor and Biomedicine, Hangzhou 310018, China

**Keywords:** neurodegenerative diseases, cGAS-STING, PANoptosis, neuroinflammation, cell death

## Abstract

**Highlights:**

**What are the main findings?**

- The cGAS-STING pathway may be associated with panoptosis, potentially regulating cell death.
- The theoretical basis for the cGAS-STING-PANoptosis axis in neurodegenerative diseases.

**What are the implications of the main findings?**

- This cellular regulatory mechanism hypothesis holds promise as a novel therapeutic approach for diseases.
- Neurodegenerative diseases face numerous safety challenges, necessitating focused attention on clinical translation and biomarkers.

**Abstract:**

Neurodegenerative diseases share features of neuronal loss, neuroinflammation, and protein aggregation. The cGAS-STING pathway, a key DNA sensor, mediates neuroinflammation via TBK1-IRF3 and IKK-NF-κB axes, inducing type I interferons and pro-inflammatory cytokines. This pathway upregulates ZBP1, promotes PANoptosome assembly, and triggers PANoptosis, releasing DAMPs and creating a self-amplifying “inflammation–death” cycle. In Alzheimer’s, Parkinson’s, and amyotrophic lateral sclerosis, pathological proteins (Aβ, Tau, α-synuclein, TDP-43) or genetic defects (e.g., C9orf72 repeats) cause mitochondrial DNA leakage or genomic instability, activating this axis. This review highlights the cGAS-STING-PANoptotic cascade as a shared pathogenic mechanism and discusses the current evidence and remaining challenges in confirming this hypothesis.

## 1. Introduction

Neurodegenerative diseases (NDDs), including Alzheimer’s disease (AD) [1], Parkinson’s disease (PD) [2], and amyotrophic lateral sclerosis (ALS) [3], are characterized by the irreversible loss of neurons, chronic neuroinflammation, and the aggregation of misfolded proteins, resulting in significant cognitive and physical impairment [4]. Despite their varied etiologies, a mounting consensus suggests that persistent, uncontrolled inflammatory responses within the brain have a key role in the onset and progression of these diseases [5].

In this context, the cyclic guanosine monophosphate-adenylate cyclase-stimulin-like protein (cGAS-STING) signaling pathway has received substantial attention for its central role in linking intracellular stress to innate immune responses [6]. As a cytoplasmic DNA sensor, cGAS exhibits elevated sensitivity to methylation of mtDNA, which serves as an efficient activator of cGAS due to its unmethylated CpG motifs [7]. The downstream effector protein STING subsequently initiates the transcription of type I interferons and numerous pro-inflammatory cytokines, thus functioning as a central regulator of both anti-infective and autoimmune responses [8].

How does this cGAS-STING-mediated chronic inflammatory signaling ultimately lead to irreversible neuronal death? PANoptosis represents a recently characterized form of programmed cell death that involves the coordinated activation and interaction of pyroptosis, apoptosis, and necroptosis [9]. The central mechanism is the formation of PANoptosome complexes, which are multiprotein platforms assembled from key molecules across multiple death pathways, including RIPK1, RIPK3, caspase-8, NLRP3, and ASC. Researchers have identified four distinct PANoptosome complexes. Each complex forms through the assembly of specific sensors and regulatory molecules. Among these, the ZBP1-PANoptosome has been most extensively studied, while evidence for the other complexes in neurodegenerative diseases remains to be further established [10,11,12].

Type I interferons generated by cGAS-STING activation have been shown to induce the expression of the key adaptor protein ZBP1. ZBP1 serves as a core platform for assembling the classical PANoptosome [13]. This suggests that the cGAS-STING pathway may be associated with panoptosis. This review thoroughly studies the key role of the emerging regulatory axis, “cGAS-STING-driven PANoptosis,” in neurodegenerative diseases. Importantly, the cGAS-STING-PANoptosis axis in neurodegenerative diseases remains an emerging hypothesis rather than a fully established pathogenic mechanism. While substantial preclinical evidence supports this system, direct evidence confirming PANoptosis in the human brain remains limited. Therefore, this review integrates current evidence to present this pathway as a working model which integrates neuroinflammation and programmed cell death.

## 2. cGAS-STING and PANoptosis Regulatory Network

The loss of neurons and glial cells in neurodegenerative diseases is a regulated process orchestrated by an elaborate molecular network. A complete understanding of the cGAS-STING pathway as an upstream regulator is essential to explain its role in integrating multiple danger signals and initiating PANoptosis, an irreversible cell death program. This basic knowledge is critical for improving the understanding of disease mechanisms.

### 2.1. Activation of CGAS

**Figure 1:**
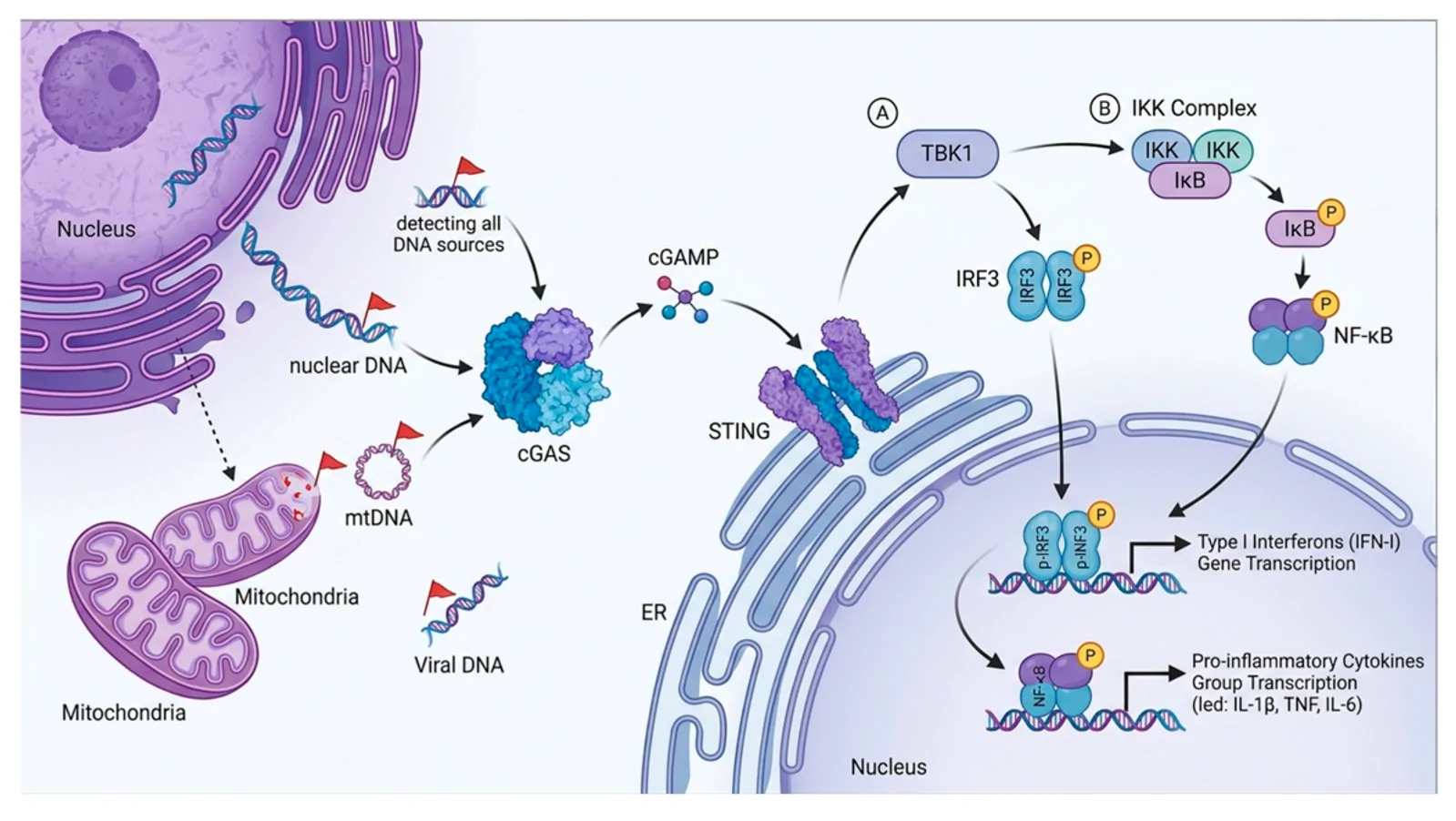
The cGAS-STING pathway-mediated innate immune activation mechanism. Multiple sources of cytoplasmic DNA—including nuclear DNA (from genomic instability), mitochondrial DNA (mtDNA, released upon mitochondrial dysfunction), and viral DNA—are recognized by the DNA sensor cGAS [6,18]. cGAS then produces the second messenger cGAMP, which binds to and activates STING anchored at the endoplasmic reticulum (ER) [8]. Activated STING simultaneously triggers two downstream signaling branches: the IRF3 pathway, which leads to type I interferon (IFN-I) gene transcription [27], and the NF-κB pathway, which drives pro-inflammatory cytokine gene transcription [28]. Both transcription factors translocate to the nucleus to initiate gene expression, collectively orchestrating innate immunity.

Genomic DNA released by viruses or intracellular bacteria during their replication cycle, or cDNA produced by reverse transcription, serves as the classic exogenous activation signal for cGAS (Figure 1) [14]. In central nervous system infections, this directly triggers a strong antiviral immune response and cell death to eliminate the infectious agent. Postherpetic neuralgia (PHN) is associated with neuroinflammation triggered by chronic varicella-zoster virus (VZV) infection. HSV-1 infection upregulates Prmt6 expression, which, in turn, methylates the STING protein, thereby blocking TBK1 and IRF3 phosphorylation and reducing type I interferon (IFN-I) production [15,16]. The suppression of the cGAS-STING pathway is a critical mechanism that enables the virus to evade immune clearance and induce postherpetic neuralgia. In monocyte-derived dendritic cells (MDDCs) and macrophages, the Non-POU domain-containing octamer-binding protein (NONO) activates the cGAS-STING signaling pathway by recognizing HIV capsid structures that have entered the cell nucleus, consequently boosting the antiviral innate immune response. Conversely, silencing NONO or cGAS significantly suppresses HIV-induced immune responses [17]. This split illustrates that the virus–host interplay at the cGAS level is highly pathogen-specific, and the same pathway can be either exploited or inhibited depending on the viral life cycle and the infected cell type.

Endogenous stress plays a more fundamental and persistent role in neurodegenerative diseases (Figure 1). Mitochondria maintain cellular energy and metabolic function and have a vital role in cellular senescence, apoptosis, and inflammatory responses. Mitochondrial dysfunction can trigger an inflammatory response, with damaged mitochondria releasing mtDNA. MtDNA enters the cytoplasm via pathways such as the BAK/BAX pore and VDAC channels, activating the cGAS-STING pathway [18]. This activation drives Type I interferon (IFN-β) and NF-κB signaling, consequently promoting neuroinflammation [19]. MtDNA is a primary endogenous danger signal in conditions such as postoperative cognitive dysfunction (POCD), where sevoflurane-induced DRP1-dependent mitochondrial fission opens mPTP-VDAC and releases mtDNA [20]. In the context of cellular senescence and autoimmunity, mitochondrial oxidative stress (e.g., reactive oxygen species [ROS]) triggers imbalances in cellular redox signaling and metabolic dysregulation [21]. Oxidative stress induces mitochondrial DNA mutations and impairs energy metabolism [22]. Autophagy, integral to cellular homeostasis, plays a central role in eliminating damaged mitochondria and preventing mtDNA leakage. However, defects in PINK1/Parkin-mediated mitophagy lead to mtDNA accumulation and subsequent cGAS-STING-driven senescence-associated secretory phenotype (SASP) [23]. Moreover, autophagy degrades cytosolic DNA and even cGAMP, constraining excessive immune responses [24]. CGAS-STING can stimulate lysosomal biogenesis via TFEB independently of TBK1, promoting pathogen clearance [25]. Under chronic neurodegenerative stress, cells must balance these antagonistic functions.

Beyond mtDNA, retrotransposon-derived DNA and p53-mediated surveillance add further layers of complexity. The wild-type p53 (WTp53) protein reduces TREX1 activity, causing the accumulation of single-stranded DNA (ssDNA) derived from retrotransposons. Cytoplasmic DNA accumulation activates the cGAS-STING pathway, which in turn drives inflammation and apoptosis. WTp53 activates TBK1 independently of MAVS (mitochondrial antiviral signaling protein), indicating its specific targeting of DNA-sensing pathways [26]. However, the role of retrotransposons is just emerging, and whether WTp53 dysfunction or TREX1 alterations contribute to chronic neuroinflammation remains entirely unknown.

### 2.2. Activation and Signal Divergence of STING

cGAS employs ATP and GTP to synthesize the novel cyclic dinucleotide cGAMP (2′–5′), which exhibits a substantially augmented capacity to activate STING—particularly in human cells—compared to its classical 3′–5′-linked isomer [29]. dsDNA instigates cGAS to form liquid–liquid phase separation aggregates, therefore enhancing cGAS activity and counteracting DNA degradation [30]. Upon activation, STING oligomerizes and forms membrane-associated aggregates in the endoplasmic reticulum, thereby constraining excessive activation [31]. Subsequently, STING complexes encapsulate cGAMP and transport it along the microtubule network from the endoplasmic reticulum through the Golgi apparatus to perinuclear compartments [32], where they recruit TBK1 and phosphorylate IRF3, thereby initiating the transcription of inflammatory mediators such as type I interferons. Non-canonical activation pathways [33]. Endoplasmic Reticulum stress plays a critical role, and pathogen metabolites, including bacterial CDN, can directly activate STING.

At the final relay point, activated STING functions as a multifunctional signaling platform (Figure 1). Within the TBK1-IRF3 pathway, STING recruits and activates the kinase TBK1 [34], which subsequently phosphorylates the transcription factor IRF3. TET-treated non-small cell lung cancer (NSCLC) cells exhibit cytoplasmic accumulation of double-stranded DNA (dsDNA), upregulation of p-STING, p-TBK1, and p-IRF3 protein expression, increased gene and protein expression of secreted chemokines CCL5 and CXCL10, and recruitment and activation of T cells and dendritic cells (DCs) [35]. Phosphorylated IRF3 dimerizes and translocates to the nucleus, driving robust expression of type I interferons (IFN-α/β) and a range of interferon-stimulated genes (ISGs) [27]. In the IKK-NF-κB pathway, NF-κB is anchored in the cytoplasm by binding to the inhibitory protein IκB. IκB masks its nuclear localization signal (NLS), thereby preventing it from entering the nucleus and binding to DNA.STING signaling stimulates proinflammatory cytokines (e.g., TNF and IL-1) to activate the IκB kinase complex (IKK), leading to phosphorylation and ubiquitin-mediated degradation of IκB. This releases NF-κB into the nucleus, where it functions as a regulatory element in gene expression [28,36]. Upon entering the nucleus, NF-κB initiates the transcription of numerous proinflammatory cytokines, such as IL-6 and TNF-α, and chemokines.

The cGAS-STING pathway mediates the production of classical type I interferons (IFN-I) and inflammatory cytokines. Beyond this primary function, the pathway also regulates a multitude of non-classical cellular functions. STING activation increases lysosomal permeability, activates the NLRP3 inflammasome, and ultimately results in STING degradation within lysosomes [37]. STING plays a key role in eliminating pathogens and exogenous nucleic acids through non-canonical autophagy pathways by promoting V-ATPase assembly via oligomerization and recruiting ATG16L1 to initiate autophagy (ATG5-dependent LC3 phosphorylation) [38]. Furthermore, STING induces DNA damage-associated senescence phenotypes and activates selective translational programs through the STING-PERK-eIF2α pathway [39].

A variety of post-translational modifications regulate the cGAS-STING pathway. For instance, the ubiquitination of TRIM30α enhances cGAS dimerization and promotes activation; USP13 removes STING ubiquitination and inhibits activation; and palmitoylation of ZDHHC1 promotes STING activation [40]. In neurodegenerative environments, chronic, low-level cGAS-STING activation triggered by endogenous DNA leads to persistent IFN-I signaling and an inflammatory cytokine environment in the brain—a state termed “type I interferon disease.” This directly impairs neuronal function and alters glial cell expression patterns [41,42].

### 2.3. Assembly of the PANoptosome

The process by which an inflammatory signaling pathway is translated into a command for cell death is an active area of research. A comprehensive review of the extant literature suggests that the cGAS-STING pathway does not directly execute cell death. Instead, it creates an environment that promotes PANoptosome assembly—a “death switch” complex that determines cellular fate—through its downstream products [43].

The primary mechanism by which the cGAS-STING pathway activates PANoptosis is via Z-DNA-binding protein 1 (ZBP1). cGAS-STING activation leads to the production of IFN-I, which, via IFNAR signaling, upregulates the expression of core PANoptosome components such as ZBP1, Caspase-8, and RIPK3 [44]. During embryogenesis, the absence of Rpa1 causes widespread DNA damage and activates the cGAS-STING pathway, driving ZBP1 transcription. Rpa1 deficiency results in the accumulation of Z-DNA bound to ZBP1, thereby triggering full ZBP1 activation. This, in turn, has been observed to induce mesenchymal stem cell death via PANoptosis [45]. Mechanistically, STING is essential for ZBP1-mediated PANoptosis (an inflammatory programmed cell death pathway) in microglia and macrophages [46]. ZBP1 is capable of recognizing viral Z-RNA (produced by DNA/RNA viruses) and endogenous transcripts (such as endogenous retrotransposons) by identifying the left-handed Z-form conformation of nucleic acids via its Zα domain. It is a core component of various PANoptosomes [47]. In the context of viral infections, cGAMP, TNF-α, and IFN-β, secreted by SARS-CoV-2-infected cells, have been shown to induce ZBP1-dependent PANoptosis in surrounding uninfected cells. This process contributes to persistent inflammation and tissue damage [48].

**Figure 2:**
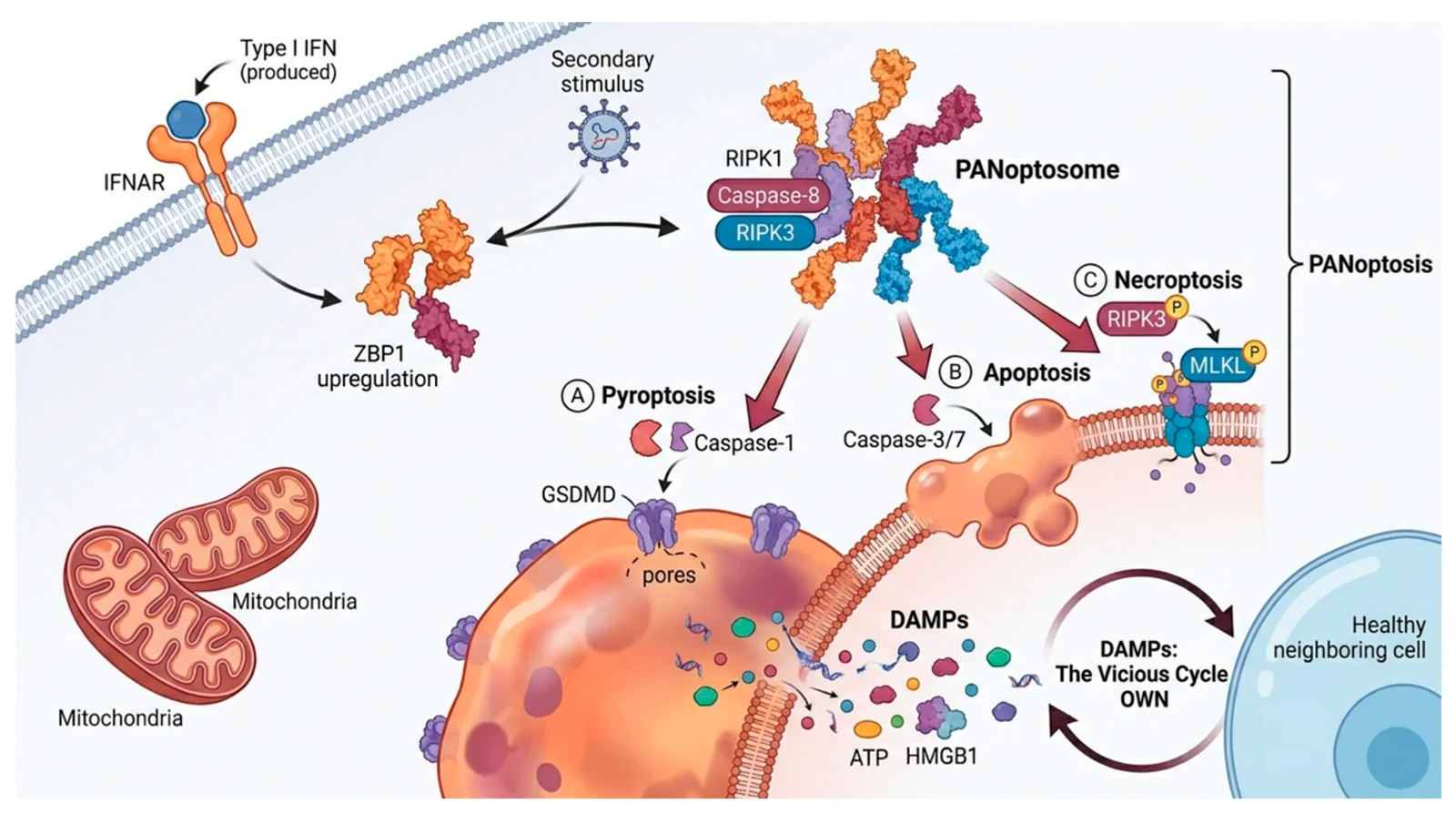
cGAS-STING-ZBP1-mediated PANoptosis and Its Positive Feedback Mechanism in Neuroinflammation. Type I interferons produced by the cGAS-STING axis, together with secondary stimuli (e.g., DAMPs/PAMPs), upregulate ZBP1 via IFNAR signaling and activate RIPK1 [44]. ZBP1 assembles with Caspase-8 and RIPK3 to form the PANoptosome complex, which simultaneously triggers three programmed cell death pathways [51]: (A) pyroptosis (Caspase-1 → GSDMD pore formation), (B) apoptosis (Caspase-3/7 activation), and (C) necroptosis (RIPK3-MLKL membrane disruption). These three pathways collectively execute PANoptosis. Dying cells release abundant DAMPs, including mtDNA, ATP, and HMGB1, which act on healthy neighboring cells to reactivate cGAS-STING and other inflammatory sensors, consequently establishing a self-amplifying vicious cycle that perpetuates neuroinflammation and neurodegeneration [54].

During PANoptosome assembly, ZBP1 functions as a scaffold protein, recruiting receptor-interacting protein kinases 1 and 3 (RIPK1, RIPK3) through its RHIM domain, as these kinases also possess RHIM domains [49] (Figure 2). Concurrently, RIPK1 suppresses ZBP1-mediated necrotic apoptosis through its RHIM domain. The generation of RIPK1mRHIM/mRHIM mutant mice, which possess inactivated RHIM domains, resulted in perinatal mortality. The lethal phenotype depends on the ZBP1/RIPK3/MLKL pathway. Complete knockout of RIPK3, MLKL, or ZBP1 rescues the lethality observed in RIPK1mRHIM/mRHIM mice [50]. This finding suggests that RIPK1 may function as a regulatory mechanism, selectively blocking the interaction between ZBP1 and RIPK3 through its RHIM domain. This, in turn, prevents excessive activation of necrotic apoptosis. In the absence of the RIPK1-RHIM domain, ZBP1 is released and subsequently triggers the RIPK3-MLKL necrotic apoptosis pathway, leading to inflammatory cell death [51] (Figure 2). Upon activation, ZBP1 triggers the assembly of the NLRP3 inflammasome, activates caspase-1 to cleave GSDMD, inducing pyroptosis (formation of pores in the cell membrane), and promotes the maturation and release of IL-1β/IL-18 [52]. Additionally, ZBP1 is regulated by multiple mechanisms: RF1 has been shown to regulate ZBP1 expression, thereby increasing NLRP3 inflammasome activity. RIPK1 has been shown to maintain homeostasis by inhibiting ZBP1-mediated necroptotic apoptosis, and NLRP3 phosphorylation/SUMOylation has been identified as a major regulator of inflammasome activity. ADAR1 has been observed to block spontaneous activation of the ZBP1-RIPK3 pathway by editing endogenous dsRNA, thereby inhibiting programmed necrosis and autoinflammation [53].

The cGAS-STING pathway exhibits positive feedback with PANoptosome assembly. For instance, inflammatory mediators produced by STING activation have been shown to induce ZBP1-mediated responses [55]. The STING-induced inflammatory environment may modify PANoptosome component function by altering RIPK1 or NF-κB activity, accordingly promoting their aggregation [56]. The KAE inhibitor suppresses the expression of critical proteins, including cGAS, STING, p-TBK1, and NF-κB. This suppression occurs by interfering with the ZBP1/cGAS-STING axis, thereby preventing PANoptosome assembly [57]. Conversely, certain PANoptosome components may also regulate STING activity. ZBP1 recognizes telomere-damage-induced TERRA via its Zα2 domain, becomes activated, and translocates to mitochondria to activate the cGAS-STING pathway. This process has been shown to drive type I interferon signaling and RIPK3-dependent necrotic apoptosis [58]. Necroptosis is not simply a downstream event of STING; rather, it involves the RIPK3–MLKL signaling axis in boosting STING pathway activity. Specifically, RIPK3 maintains sustained STING activation by inhibiting its autophagic degradation, while MLKL amplifies the STING signal through a membrane pore-dependent mechanism; together, these two components form a positive feedback loop [59]. ZBP1 and cGAS-STING interact during viral invasion in innate immunity, tightly coupling innate immune sensing with cell death execution. ZBP1 enhances the STING-TBK1-IRF3 axis; however, viruses can cleave cGAS, thereby inhibiting this pathway. Its precise regulation functions as an essential target for balancing pathogen clearance and inflammatory damage [60]. It is imperative to ensure that cells can initiate a complete death process to protect the body when threats exceed the scope of a controllable inflammatory response.

Upon PANoptosome assembly, it activates three downstream cellular death pathways. These effects result in a rapid and complete loss of cell membrane integrity. This results in the violent release of cellular contents, including large quantities of DAMPs such as HMGB1, ATP, mtDNA, IL-1α, and others. The DAMPs released further activate the cGAS-STING pathway, the NLRP3 inflammasome, and ZBP1 in surrounding neurons and glial cells [54]. This forms a self-amplifying “inflammation-death” vicious cycle, which may play a central role in the spatial expansion and chronic progression of neurodegenerative diseases. However, the precise molecular determinants that shift the cGAS-STING response from protective homeostatic to pathogenic PANoptotic remain elusive.

## 3. Specific Mechanisms in Neurodegenerative Diseases

**Figure 3:**
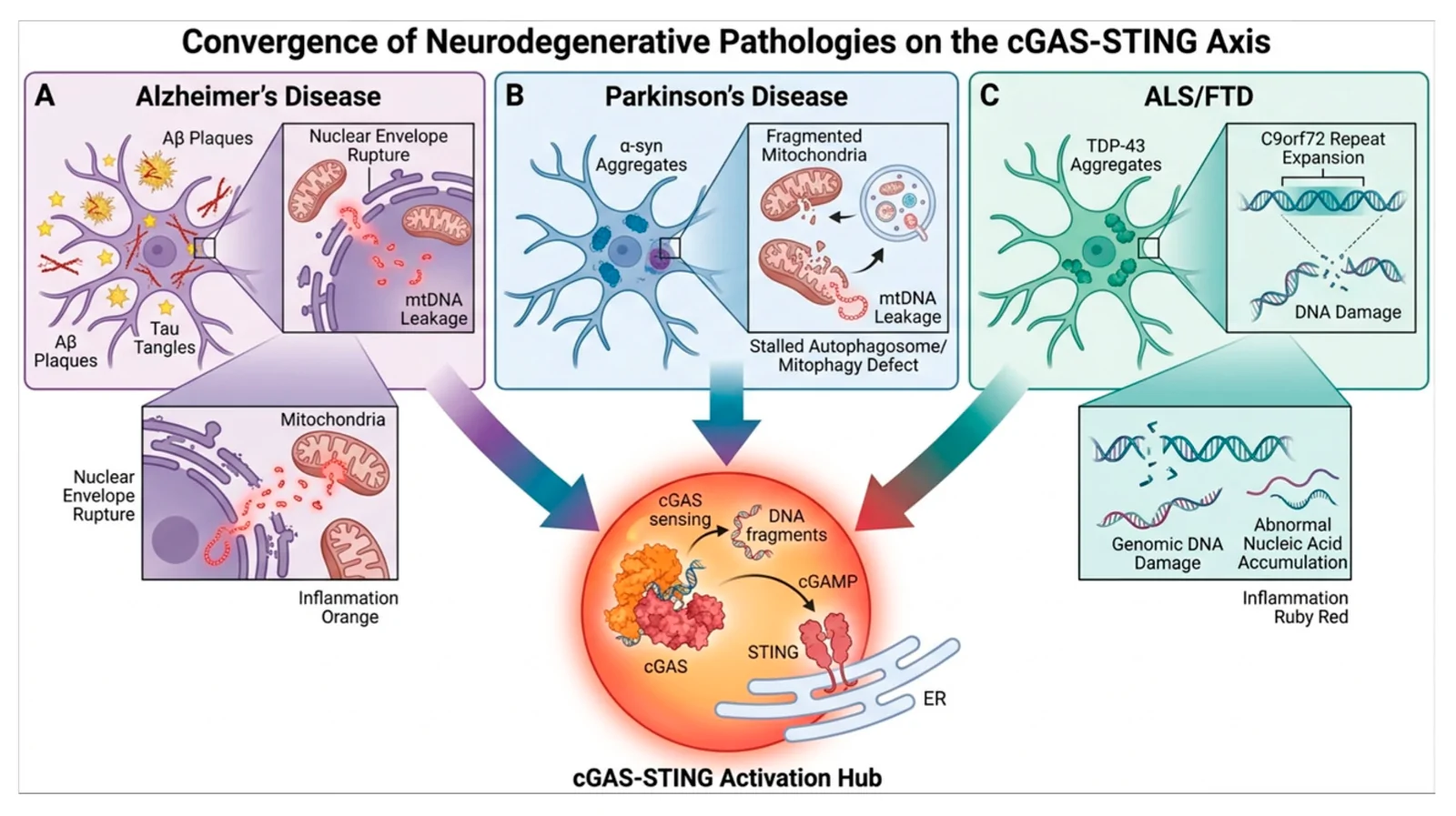
The shared cGAS-STING inflammatory core pathway in various neurodegenerative diseases. Three major neurodegenerative diseases—Alzheimer’s disease (AD), Parkinson’s disease (PD), and amyotrophic lateral sclerosis/frontotemporal dementia (ALS/FTD)—are depicted with their respective upstream pathological events. In AD (A), Aβ plaques, Tau tangles, nuclear envelope rupture, and mtDNA leakage lead to cytoplasmic DNA accumulation [61]. In PD (B), α-synuclein aggregates, fragmented mitochondria, stalled mitophagy/autophagy, and DNA damage result in mtDNA and genomic DNA release [62]. In ALS/FTD (C), TDP-43 aggregates, C9orf72 repeat expansion, nuclear envelope rupture, and abnormal nucleic acid accumulation generate diverse cytoplasmic nucleic acid species [63,64]. All these upstream events converge on the cGAS-STING activation hub, where the DNA sensor cGAS recognizes mtDNA, genomic DNA fragments, and abnormal nucleic acids, produces cGAMP, and activates STING at the endoplasmic reticulum (ER) [33]. The hub ultimately outputs inflammatory signals (type I interferons and pro-inflammatory cytokines), illustrating that despite distinct initiators, these diseases share a common core inflammatory pathway [65,66,67].

The aforementioned cGAS-STING-PANoptosis regulatory network is not simply theoretical speculation. The hypothesis has gained increasing support from preclinical and clinical evidence in major neurodegenerative diseases, such as Alzheimer’s disease, Parkinson’s disease, and amyotrophic lateral sclerosis (ALS) [40]. This section explains how this core axis is activated and drives pathological processes across multiple disease contexts, revealing its central role as a common pathway (Figure 3).

### 3.1. Alzheimer’s Disease

The hallmark pathological features of Alzheimer’s disease (AD) are extracellular amyloid beta (Aβ) plaques and intracellular neurofibrillary tangles [1]. Mitochondrial dysfunction provides the strongest current link between AD and cGAS-STING activation (Figure 3A). Aβ oligomers disrupt the mitochondrial respiratory chain and release mtDNA, activating cGAS [68,69]. This exacerbates Aβ plaque deposition [70], neuroinflammation, pyroptosis, and impaired autophagy function [71]. In addition, intracellular accumulation of β-amyloid also disrupts lysosomal membranes, leading to leakage of phagocytosed DNA-like substances [72]. However, the question of which—mtDNA or lysosomal DNA—dominates in neurons and microglia, as well as whether their relative contributions vary throughout various disease stages, remains unanswered. Evidence linking Tau pathology to cGAS-STING activation is more indirect than that for Aβ. Hyperphosphorylated tau may promote nuclear envelope disruption [73], and glycated tau can destabilize heterochromatin [74], potentially exposing genomic DNA to cGAS, although direct demonstration in human AD neurons is lacking.

The interaction between AD and cGAS-STING is reflected in protein expression levels. In postmortem AD brains and APP/PS1 mice, cGAS and STING protein levels and their phosphorylation markers (p-TBK1, p-IRF3) are elevated and correlate with pathology [65,75]. More direct evidence comes from genetic and pharmacological interventions. CGAS deficiency reduces inflammation [76], and agents that stabilize mitochondria (honokiol [77], nicotinamide riboside [78]) suppress cGAS-STING and improve cognition. In AD, elevated expression levels of various PANoptosis-related molecules (ZBP1, RIPK1, RIPK3, MLKL, and GSDMD) have also been detected [79], and cell death cannot be fully blocked by single-pathway inhibitors, but is mitigated by ZBP1 or caspase-8 targeting [80,81]. This is consistent with PANoptosis, although direct demonstration of the PANoptosome complex in human AD neurons is lacking.

### 3.2. Parkinson’s Disease

The fundamental pathology of Parkinson’s disease (PD) is characterized by the degeneration of dopaminergic neurons in the substantia nigra pars compacta, accompanied by the accumulation of Lewy bodies (rich in α-synuclein) [2]. These dopaminergic neurons are highly sensitive to mitochondrial dysfunction and cGAS-STING activation (Figure 3B).

Neuroinflammation induced by microglial activation and impaired neuronal mitochondrial phagocytosis are considered key pathogenic mechanisms in Parkinson’s disease (PD) [62]. RIPK1 promotes glial cell-mediated neuroinflammation by regulating the necroptotic axis (RIPK1-RIPK3-MLKL), proinflammatory signaling (NF-κB/MAPKs), and antioxidant pathways (Nrf2/CREB) [82]. The targeted inhibition of RIPK1 (Nec-1/Nec-1s) significantly alleviates acute LPS-induced inflammation and neurodegenerative pathology in MPTP-induced PD models [83]. Pharmacological activators of the PINK1/Parkin mitophagy pathway (e.g., β-asarone, urushiol) show neuroprotective potential [84]. Paradoxically, MLKL deficiency exacerbates neurodegeneration in α-synuclein transgenic models by blocking inflammatory microglial activation [85], underscoring the need for cell-type-specific and temporal analyses.

The effects of α-synuclein aggregates on PD are direct [86]. The induction of S-nitrosylation modification of the Parkin protein by α-synuclein oligomers can inhibit its E3 ubiquitin ligase activity. This results in defective mitophagy, the accumulation of damaged mitochondria, reduced mitochondrial biogenesis (downregulation of PGC-1α), and disrupted mitochondrial dynamics (enhanced Drp1-dependent fission) [87]. This leads to mtDNA release and cGAS-STING activation in microglia [66,88]. However, it remains unclear whether α-synuclein directly activates cGAS or acts solely through mitochondrial damage.

The study determined that the accumulation of DNA double-strand breaks (DSBs) and/or DNA repair defects constitutes a key mechanism in the pathogenesis of Parkinson’s disease [89]. In the MPTP mouse model, cGAS in microglia activates the STING pathway by sensing damaged DNA, driving the release of inflammatory factors such as IFNβ and CXCL10 and leading to dopaminergic neuronal death and progression of Parkinson’s disease [90]. Inhibition of the STING pathway has been shown to alleviate α-Syn-mediated neuroinflammation and degeneration [91]; however, further research is necessary to validate this finding in human disease models. Most evidence derives from acute toxin models (MPTP, LPS) or α-Syn-PFF injection, which recapitulate aspects but not the full chronic, age-dependent progression of human PD.

### 3.3. Amyotrophic Lateral Sclerosis

Amyotrophic lateral sclerosis (ALS) is a neurodegenerative illness characterized by the progressive death of motor neurons. It is associated with cytoplasmic aggregation of TDP-43 protein, amplification of repetitive DNA sequences, and severe DNA damage responses (Figure 3C) [3]. TDP-43 provides the most direct mechanistic link: cytoplasmic aggregates infiltrate mitochondria via TIM22/TOM20, triggering mPTP opening and mtDNA release, which activates cGAS-STING [63,67,92]. STING inhibition ameliorates pathology [67]. Nuclear TDP-43 loss-of-function may add via impaired DNA repair [93]. VAPB-mediated STING regulation [94] adds complexity, but its role in ALS pathogenesis is less established.

The most common genetic cause of amyotrophic lateral sclerosis (ALS) is an expansion of the GGGGCC hexanucleotide repeat in the C9orf72 gene. C9orf72 repeats activate cGAS through multiple proposed mechanisms, including R-loops, DPR toxicity, and fragile-site-induced micronuclei [64,95]. However, the relative contribution of each pathway in human ALS remains unknown. Overexpression of C9orf72 can alleviate DSS-induced ulcerative colitis inflammation and intestinal barrier damage by inhibiting the cGAS-STING signaling pathway [96].

Furthermore, in aging and stressed motor neurons, retrotransposon activity, such as LINE-1, increases, and the cDNA they produce acts as a potent cGAS agonist [97]. Concurrently, cGAS in the nucleus imposes limitations on L1 retrotransposition [98]. These mechanisms help ensure genomic stability within the DNA damage response and in the context of cellular aging. Pro-inflammatory signals can spread to neighboring glia [99], and type I/II IFN signatures differ between mice and humans [100]. Motor neuron death involves caspase-dependent and MLKL-mediated pathways, and GSDME has been implicated [101,102]. However, it is unclear whether these effectors are activated in parallel or sequentially, and what upstream signals determine pathway choice.

### 3.4. Cross-Disease Commonality

Although the triggering factors differ in focus (Aβ/Tau, α-syn, TDP-43), these diseases share a core vicious cycle driven by the cGAS-STING-PANoptosis axis [61,67,91]. In AD, Aβ oligomers induce lysosomal membrane permeabilization and mitochondrial dysfunction, with mtDNA serving as the primary cGAS agonist [68]. In PD, α-synuclein aggregates impair mitochondrial complex I and disrupt PINK1/Parkin-mediated mitophagy, leading to mtDNA accumulation and STING-dependent neuroinflammation [87]. In ALS/FTD, TDP-43 directly invades mitochondria via TIM22/TOM20 translocases to trigger mtDNA release through mPTP/VDAC, while C9orf72 hexanucleotide repeat expansions generate R-loops and RNA-DNA hybrids that also activate cGAS [63,64].

Critical evidence gaps and still unresolved controversies remain. The temporal dynamics of cGAS-STING activation differ across diseases: AD and PD feature prolonged prodromal phases, whereas ALS progresses more rapidly [4]. Furthermore, all preclinical and clinical trials based on exogenous neurotoxins to date have failed; a new concept of neurodegeneration in Parkinson’s disease based on single-neuron degeneration has even been proposed [103].

## 4. Therapeutic Targets and Intervention Strategies

Given the central role of the cGAS-STING-PANoptosis axis in driving the progression of neurodegenerative diseases, as previously explained, targeting its components has emerged as a highly promising new therapeutic strategy. This chapter will methodically review possible therapeutic targets, from upstream signaling sources to downstream effectors, and assess their translational prospects and challenges (Table 1).

### 4.1. Inhibition of cGAS-STING Pathway Activation

Inhibiting the cGAS-STING pathway is the most direct upstream intervention strategy, aimed at suppressing inflammatory signal generation at the source. Upstream inhibition offers the broadest effect but highest immune risk. The natural compounds thymolyl alcohol (PAH) and EGCG can either disrupt the G3BP1–cGAS complex or inhibit mtDNA release. These compounds represent a novel mechanism with high safety profiles, yet they exhibit multi-target effects and low bioavailability [104]. Additionally, sulfonamide drugs and chloroquine derivatives can interfere with the interaction between cGAS and dsDNA; among these, chloroquine derivatives have a solid clinical basis but poor specificity, whereas sulfonamide derivatives are more potent and specific, though most remain in the preclinical stage [108].

The cGAS inhibitors currently under investigation interfere with downstream reactions by competitively binding to cGAS’s catalytic active site. Small-molecule inhibitors such as RU.521 [105], G140 [106], and G150 [126] effectively inhibit cGAS activity in cellular and animal models [107]. RU.521 is the preferred investigational drug for preclinical studies in mice, whereas G140 and G150 are more promising candidates for studies involving human cells or for developing human therapeutic agents. However, this approach may alter cGAS’s physiological role in detecting pathogen DNA, and long-term systemic inhibition of cGAS may increase infection risk. Developing brain-targeted delivery systems or conditional-knockout strategies is imperative to improve safety.

STING inhibitors—C-176 [127], H-151 [109], Astin C [128] and the non-covalent highly selective inhibitor SN-011 [110]—prevent STING oligomerization, Golgi transport, and TBK1-IRF3 recruitment. Among these, SN-011 is particularly suitable for human cell-based assays due to its high selectivity and low toxicity [129]. Disulfiram (DSF) offers an alternative mechanism, targeting RNF115 to impede K63-ubiquitination-dependent STING activation [111] and inhibiting NLRP3 palmitoylation [112]. In addition to directly interfering with STING, another potential approach is to target the cGAMP signaling pathway. This could be done by developing activators or mimetics of cGAMP-degrading enzymes (such as the extracellular protease ENPP1), or by using competitive cGAMP antagonists. Exosomes expressing ENPP1 hydrolyze cGAMP, block the STING-IRF3 pathway, and inhibit type I interferon production [113].

### 4.2. Targeting Key Nodes of PANoptosis

In the downstream sequence of cGAS-STING, selective interference with PANoptosome assembly or function can block inflammatory cell death whilst preserving some upstream immune signaling functions. Midstream targeting may preserve more physiology but requires proof that PANoptosis is the dominant death modality; downstream blockade is simple but risks interfering with essential apoptosis.

Functioning as a core hub linking the cGAS-STING signaling pathway and PANoptosis, ZBP1 is a highly attractive target. Although clinical-grade small-molecule inhibitors are currently lacking, gene knockdown/knockout studies have shown potent protective effects in various models, including viral infection and sepsis [130]. A short isoform, ZBP1-S, functions as an endogenous inhibitor by competitively binding Z-DNA [114], and ADAR1 engages ZBP1’s Zα2 domain to impede RHIM–RIPK3 binding [131]. Scutellarin curtails macrophage PANoptosis by preserving mitochondrial function, blocking ZBP1-PANoptosome assembly, and suppressing pyroptosis, apoptosis, and necroptosis markers simultaneously [115]. RIPK1 inhibition (Nec-1s, GSK’872, RIPA-56) [116,119] has shown neuroprotective effects in PD, AD, and cerebral ischemia models. Nec-1s mitigates neuronal death and amyloid/Tau pathology and reverses the pro-inflammatory microglial phenotype [117]. DNL747, a RIPK1 inhibitor, entered early clinical trials for neurodegenerative diseases but was terminated due to dose-limiting toxicity (anemia/thrombocytopenia) in long-term nonclinical studies [118]. Targeting PANoptosome assembly has the theoretical advantage of preserving upstream cGAS-STING antiviral functions while specifically blocking pathological cell death. This midstream intervention delivers a more targeted approach than upstream inhibition with potentially fewer off-target effects. However, this approach confronts multiple challenges. PANoptosome assembly involves multiple components (ZBP1, RIPK1, RIPK3, caspase-8, NLRP3, ASC); targeting a single component may be insufficient if the complex can assemble through alternative routes.

Targeting common downstream effectors—caspases (apoptosis/pyroptosis) and GSDMD (pyroptosis). Broad-spectrum caspase inhibitors (Q-VD-OPh) [120] and selective caspase-1 inhibitors (VX765) [121] have shown preclinical efficacy. VX765 inhibits LPS+ATP-induced caspase-1 activation, IL-1β release, and GSDMD cleavage, blocking pyroptosis [122]. GSDMD inhibitors (disulfiram, necrotic sulfonamide) [124] have shown preliminary success in neuroinflammation models; disulfiram significantly alleviates neuroinflammation, neuronal damage, and behavioral deficits in PD models [125]. This approach carries the greatest risk of interfering with physiological apoptosis, which is essential for development and tissue homeostasis. Broad-spectrum caspase inhibitors may have significant side effects with long-term use. When TLR3/4 ligands synergize with pan-caspase inhibitors, they may paradoxically enhance ROS levels, induce mitochondrial damage, and drive macrophage death through alternative pathways [123]. Moreover, the therapeutic window for downstream inhibitors is narrow, and their efficacy rests on accurate patient stratification based on the predominant death pathway.

## 5. Challenges and Prospects

This study is pioneering in systematically constructing the cGAS-STING pathway and PANoptosis as a unified “perception-death” regulatory axis in neurodegenerative diseases. Mechanistically, cGAS-STING produces type I interferons and inflammatory factors via two main pathways: TBK1-IRF3 [35] and IKK-NF-κB [110]. These pathways collaborate to upregulate key molecules, such as ZBP1. GSDMD activation initiates the process, which subsequently triggers caspase assembly and MLKL release [132]. This cascade ultimately releases damage-associated molecular patterns (DAMPs).

Despite the encouraging outlook, therapeutic development in this domain continues to encounter major challenges. The cGAS-STING pathway plays a critical physiological role in anti-infection and antitumor responses. Prolonged inhibition leads to opportunistic infections or impaired tumor surveillance [108,118]. Establishing precise therapeutic windows and dosages, along with local administration regimens for the central nervous system, is paramount [133]. This axis may serve a central role in the early stages of the disease, particularly during the initiation and dissemination phase of inflammation. In advanced stages of the disease, when a significant number of neurons have already succumbed, the effectiveness of anti-inflammatory and anti-apoptotic therapies may be constrained. Consequently, treatment may require a combination with early diagnostic biomarkers. In addition, this approach manifests species-specific variations and model limitations. Most existing mouse models fail to recapitulate the key transcriptional alterations observed in human neurodegenerative diseases [134]. In vitro animal testing is limited; the probability of a new drug’s indication progressing from Phase I human trials to market approval ranges from 8% to 14%, with success rates particularly low in adult neurodegenerative diseases [135]. Consequently, confirming the occurrence of PANoptosis and quantifying its contribution to total neuronal loss remains difficult, particularly in human brains in vivo. Conventional histopathological techniques (e.g., TUNEL, cleaved caspase-3 staining) typically detect only one pathway at a time [136]; examining emerging imaging modalities or PET tracers [137] could non-invasively assess cell death activity in the brain.

Targeting the cGAS-STING-PANoptosis axis constitutes a novel paradigm for disease-modifying therapy, integrating neuroimmunology and cell death. The critical factor enabling the shift from mechanistic elucidation to clinical translation within this domain is precisely balancing the threshold between immune protection and inflammatory damage. By intervening precisely in this core vicious cycle, we are poised to develop broad-spectrum therapies capable of slowing or even halting the common progression of multiple neurodegenerative diseases.

## Figures and Tables

**Table 1:** Potential therapeutic methods targeting the cGAS-STING-PANoptosis axis.

Intervention Level	Specific Target	Representative Drugs/Methods	Mechanism of Action
cGAS-STING activation	cGAS	PAH [104], EGCG [104], RU.521 [105], G140 [106], G150 [107]	Interference with cGAS-dsDNA binding [108]; competitive binding to catalytic active sites [106]; inhibition of cGAMP synthesis [107].
	STING	C-176 [107], H-151 [109], SN-011 [110], Disulfiram (DSF) [111]	Inhibition of STING oligomerization [110], translocation, downstream kinase recruitment [110], and membrane localization (palmitoylation) [112].
	cGAMP/ENPP1	ENPP1-OE exosome [113], cGAMP antagonist [113]	Exosomes express ENPP1 to hydrolyze extracellular cGAMP, thereby interrupting signal transduction [113].
PANoptosome assembly	ZBP1	ZBP1-S [114], Scutellarin [115]	Blocking Z-DNA formation [114]; Competitive inhibition of ZBP1-PANoptosome complex assembly [115].
	RIPK1/RIPK3/MLKL	GSK’872 [116], Necrostatin-1s (Nec-1s) [117], DNL747 [118]	Inhibition of necrotic apoptosis execution complex; suppression of RIPK1/3 kinase activity [116,119].
Effector molecule blockade	Caspases	Q-VD-OPh [120], VX765 (Casp-1) [121]	VX765 can suppress NLRP3 inflammasome assembly [122]; Inhibition of apoptosis and pyroptosis execution [122,123].
	GSDMD	Disulfiram, Necrotic sulfonamide [124]	Inhibits GSDMD cleavage and pore formation, blocks inflammatory factor release and pyroptosis [125].

## Data Availability

No new data were created or analyzed in this study. Data sharing is not applicable to this article.
